# Comparative evaluation of persulfate and peroxy monosulfate-based advanced oxidation processes for amoxicillin degradation: mechanisms, efficiency, and challenges

**DOI:** 10.1039/d5ra09933a

**Published:** 2026-03-18

**Authors:** Harez R. Ahmed, Anu Mary Ealias

**Affiliations:** a Department of Chemistry, College of Science, University of Sulaimani Qlyasan Street Sulaymaniyah 46001 Kurdistan Region Iraq harez.ahmed@univsul.edu.iq; b College of Science, Department of Medical Laboratory Science, Komar University of Science and Technology 46001 Sulaimani Iraq; c Department of Civil Engineering, VIT Mauritius, Uniciti International Education Hub 72448 Pierrefonds Mauritius

## Abstract

The widespread occurrence of antibiotics in aquatic environments has raised serious concerns due to their persistence, toxicity, and contribution to the proliferation of antibiotic-resistant bacteria. Amoxicillin, one of the most frequently prescribed β-lactam antibiotics, is frequently detected in surface water, groundwater, and wastewater effluents due to its low biodegradability and incomplete removal by conventional treatment technologies. In this context, sulfate radical-based advanced oxidation processes (SR-AOPs) have emerged as highly effective alternatives for degrading antibiotics. This review critically evaluates activation-based AOPs using persulfate (PS) and peroxymonosulfate (PMS) for the degradation of amoxicillin, with emphasis on oxidant chemistry, radical generation mechanisms, redox properties, stability, and reaction kinetics. Various activation strategies, including thermal, UV/visible-light, transition-metal catalysis, carbonaceous material activation, and electrochemical/photoelectrocatalytic systems, are systematically compared with respect to efficiency, selectivity, operational conditions, and environmental implications. The advantages of sulfate radicals over hydroxyl radicals, including longer lifetimes, broader pH applicability, and enhanced selectivity for electron-rich antibiotic structures, are highlighted. Key challenges related to catalyst stability, metal leaching, energy consumption, and toxicity of transformation products are also discussed. Finally, future research directions are proposed to facilitate the scale-up and sustainable application of PS/PMS-based AOPs for treating antibiotic-contaminated water.

## Abstract 2

The widespread occurrence of antibiotics in aquatic environments has raised serious concerns due to their persistence, toxicity, and contribution to the proliferation of antibiotic-resistant bacteria.

## Graphical abstract

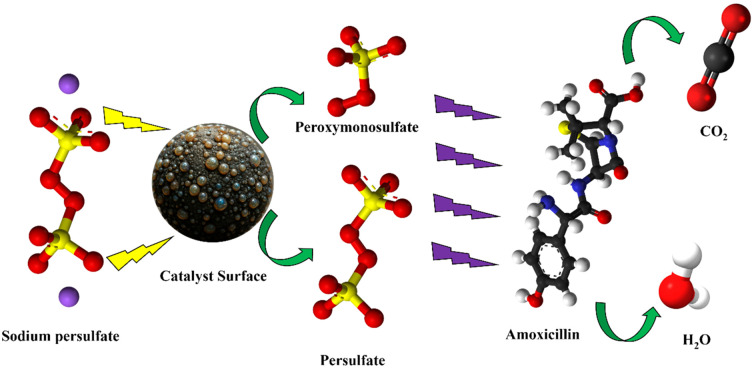

## 1. Introduction

Antibiotics have emerged as a critical class of environmental contaminants, raising global concern due to their extensive use in human medicine, veterinary practice, and animal husbandry.^1^ Large quantities of these compounds enter natural ecosystems *via* domestic and industrial wastewater effluents, where conventional treatment systems are often unable to remove them^2^ completely. Consequently, their accumulation in soil, surface water, and groundwater has escalated into a major environmental and public health issue. The World Health Organization (WHO) has identified antibiotic resistance as one of the most significant future threats to global health, driven largely by the persistent exposure of microorganisms to sub-lethal concentrations of antibiotics in the environment.^2^

A key challenge is that many antibiotics exhibit low biodegradability and high environmental persistence, allowing them to remain in aquatic systems for extended periods and bioaccumulate across various ecological compartments.^3,4^ Amoxicillin, a widely prescribed β-lactam antibiotic for bacterial infections, is of particular concern. Due to its low metabolic transformation in humans, approximately 70–90% of administered amoxicillin is excreted unchanged, ultimately reaching wastewater streams and natural waters.^5^ Chronic exposure to residual antibiotics increases selection pressure for antibiotic-resistant bacteria and resistance genes, reducing the efficacy of antimicrobial therapies and posing major risks to human and ecosystem health. Recent studies have detected amoxicillin and its degradation products even in treated drinking water, underscoring its classification as a high-priority emerging pollutant.^5–7^

Amoxicillin (AMX) is among the most widely prescribed β-lactam antibiotics globally and is frequently detected in aquatic environments due to its extensive use in human and veterinary medicine. Numerous monitoring studies have reported the presence of AMX in various environmental matrices at varying concentrations. In municipal wastewater and hospital effluents, AMX concentrations typically range from several nanograms per liter (ng L^−1^) to several micrograms per liter (µg L^−1^), depending on consumption patterns and treatment efficiency. Surface waters receiving treated or untreated wastewater discharges have also shown detectable levels of AMX, generally within the range of 10–500 ng L^−1^, while concentrations in hospital wastewater can occasionally exceed several µg L^−1^. Although lower concentrations are usually detected in groundwater and drinking water sources, the continuous release of antibiotics into aquatic environments raises concerns regarding ecological toxicity and the development of antimicrobial resistance. These findings highlight the need for advanced and efficient treatment technologies capable of removing AMX from complex wastewater matrices.

To mitigate these risks, various physical, chemical, and biological treatment technologies have been explored, such as chlorination, ultraviolet (UV) irradiation, ozonation, photocatalysis, Fenton reactions, electrocatalysis, adsorption, membrane filtration, sand filtration, and bioreactors.^8–14^ However, many of these processes exhibit low efficiency, generate toxic byproducts, have narrow pH windows of operation, or incur high operating costs. In contrast, advanced oxidation processes (AOPs) have demonstrated strong potential for degrading antibiotics by generating highly reactive species that mineralize complex organic molecules into harmless, biodegradable intermediates.^15^

Among AOPs, sulfate radical-based advanced oxidation processes (SR-AOPs) have gained significant attention due to their strong oxidation capacity and improved performance under diverse environmental conditions. Sulfate radicals (SO_4_˙^−^) possess several advantages over hydroxyl radicals (˙OH), including effective reactivity across a broad pH range (2–11), higher mineralization potential, longer half-life, greater selectivity toward electron-rich pollutants, and environmentally benign end-products (predominantly sulfate ions). Peroxymonosulfate (PMS, HSO_5_^−^) is a key precursor of sulfate radicals, which can be activated by a wide range of stimuli—including ultrasound, metal ions, UV/visible light, microwaves, carbonaceous materials, semiconductors, and thermal energy.^16–18^ PMS activation often results in the simultaneous generation of SO_4_˙^−^ and ˙OH radicals, enabling synergistic oxidation pathways. Notably, photocatalytic activation of PMS has emerged as a particularly promising strategy owing to its enhanced radical-generation efficiency and its ability to mineralize diverse organic contaminants under mild conditions.^19^

Persulfate (PS, S_2_O_8_^2−^) has also attracted increasing research interest as a robust oxidant in SR-AOPs. SO_4_˙^−^ generated from PS exhibits a high redox potential (2.6 V), a relatively long lifetime, and strong selectivity, making it comparable in oxidative strength to ˙OH (2.8 V) but with improved stability. Additional benefits of PS include its low cost, solid-state stability, ease of transport and storage, high solubility, and the ability to generate stable radicals across various environmental matrices. Persulfate can be activated by heat, UV irradiation, transition metals, or ultrasonic waves to generate sulfate radicals that can oxidize a wide range of persistent organic pollutants, including antibiotics.^20–22^ This review provides a comprehensive overview of persulfate- and peroxymonosulfate-based advanced oxidation processes for the degradation of amoxicillin in aquatic environments. First, the fundamental physicochemical properties and radical generation mechanisms of persulfate and peroxymonosulfate are discussed to clarify their roles in sulfate radical-based oxidation systems. Next, the major activation strategies, including thermal, photochemical, transition-metal, carbonaceous material, electrochemical, and photoelectrocatalytic methods, are systematically examined. The review then summarizes recent progress in the degradation pathways and reaction mechanisms involved in amoxicillin removal. In addition, the influence of real wastewater matrices on process efficiency is critically discussed to highlight practical challenges in environmental applications. Finally, current limitations, catalyst stability issues, and opportunities for future research are presented to inform the development of more efficient and sustainable sulfate radical-based treatment technologies.

## 2. Persulfate and monosulfate chemistry

Sulfate radical-based advanced oxidation processes (SR-AOPs) have been widely applied for the degradation of antibiotics in water and wastewater, demonstrating high effectiveness in treating persistent and emerging contaminants. In recent years, SR-AOPs have attracted significant attention owing to their strong oxidative capacity, operational versatility, and applicability to a broad spectrum of organic pollutants. Persulfate (PS, S_2_O_8_^2−^) and peroxymonosulfate (PMS, HSO_5_^−^) serve as the primary precursors for generating sulfate radicals (SO_4_˙^−^), which are powerful oxidants capable of mineralizing a variety of recalcitrant compounds. These oxidants can be activated *via* several pathways: thermal, alkaline, ultraviolet irradiation, activated carbon, transition metals (*e.g.*, Fe^0^, Fe^2+^, Cu^2+^, Co^2+^, Ag^+^), ultrasound, and hydrogen peroxide, each of which facilitates homolytic cleavage of the peroxy bond, generating reactive sulfate radicals. Compared with hydroxyl radical (˙OH)-based AOPs, sulfate radical systems exhibit several advantages, including a higher oxidation potential, greater selectivity, and enhanced efficiency for pollutants containing unsaturated bonds, aromatic rings, or electron-rich functional groups. Furthermore, sulfate radicals are highly reactive across a wider pH range and have longer half-lives than hydroxyl radicals in many environmental matrices, enabling more sustained oxidative activity during treatment processes.^23^ These attributes make SO_4_˙^−^ particularly effective at degrading emerging contaminants, including antibiotics, pharmaceuticals, and endocrine-disrupting compounds.

Persulfate is typically encountered as a colorless or white crystalline solid with high chemical stability and excellent water solubility (730 g L^−1^). Aqueous persulfate solutions are mildly acidic. Structurally, PS possesses a symmetrical peroxo bond with an O–O distance of 1.497 Å and a bond dissociation energy of approximately 140 kJ mol^−1^, contributing to its high stability and the need for activation. The most common laboratory forms include sodium persulfate (Na_2_S_2_O_8_) and potassium persulfate (K_2_S_2_O_8_).^24,25^ Peroxymonosulfate, by contrast, is a white solid powder with an asymmetrical molecular structure. It's O–O bond length (1.453 Å) and higher estimated bond dissociation energy (140–213.3 kJ mol^−1^) influence its reactivity and activation behavior. PMS is highly soluble in water (>250 g L^−1^), generating an acidic solution. It exhibits its greatest stability under strongly acidic conditions (pH < 6) or highly alkaline environments (pH ≈ 12), whereas at pH 9 it decomposes more rapidly, with approximately half of HSO_5_^−^ converting to SO_5_^2−^.^26,27^ Both PS and PMS are strong oxidants with standard redox potentials of 2.01 V and 1.82 V, respectively. Nevertheless, their direct reaction with organic pollutants is generally slow, necessitating appropriate activation strategies to generate the more reactive sulfate radicals (SO_4_˙^−^) and hydroxyl radicals (˙OH). Effective activation is therefore essential to harness the full oxidative potential of persulfate- and PMS-based AOPs in water treatment applications.

### 2.1 Decomposition mechanisms of persulfate and monosulfate

Persulfate and monosulfate undergo homolytic cleavage upon activation, producing radical species with strong oxidative power.

1 S_2_O_8_^2−^ → 2SO˙^−^

Persulfate contains a peroxide bond (–O–O–) with a relatively high activation energy, requiring external stimuli—such as heat, UV irradiation, transition metals (*e.g.*, Fe^2+^, Co^2+^), ultrasound, or carbon-based catalysts—to initiate homolytic bond cleavage. The resulting SO_4_˙^−^ radicals are highly oxidizing, selective toward electron-rich functional groups, and remain reactive over a wide pH range.^28^

2 HSO_5_^−^ → SO_4_˙^−^ + ·OH

**Fig. 1:**
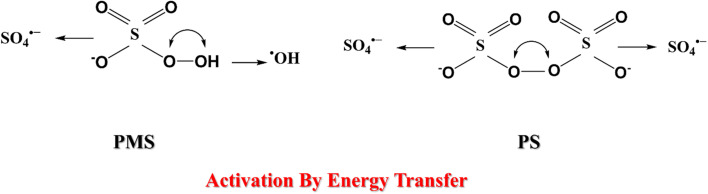
Schematic illustration of the decomposition mechanisms of persulfate (PS) and peroxymonosulfate (PMS) through energy-transfer processes leading to the generation of reactive radicals. The activation of PS produces two sulfate radicals (SO_4_˙^−^), while PMS decomposition generates both sulfate (SO_4_˙^−^) and hydroxyl (˙OH) radicals. These highly reactive species initiate electron-transfer and hydrogen-abstraction reactions responsible for the degradation and mineralization of organic pollutants.

PMS contains both peroxide and peroxyacid functionalities, enabling simultaneous formation of sulfate radicals (SO_4_˙^−^) and hydroxyl radicals (˙OH). This dual-radical pathway enhances its oxidation versatility, making PMS highly efficient even under mild conditions. UV light, transition metals, carbonaceous catalysts, ultrasound, and heat are typically used to activate PMS.^29^ The activation radicals by energy transfer are illustrated in Fig. 1.

### 2.2 Fundamental oxidation chemistry of persulfate and peroxymonosulfate in sulfate radical-based AOPs

The redox behavior, stability, and radical generation kinetics of persulfate (PS) and peroxymonosulfate (PMS) are central to understanding their efficiency in sulfate radical-based advanced oxidation processes. Both oxidants generate highly reactive species, with SO_4_˙^−^ exhibiting a redox potential of 2.5–3.1 V and ˙OH 2.7–2.8 V, while the parent oxidants S_2_O_8_^2−^ and HSO_5_^−^ possess redox potentials of 2.1 V and 1.8 V, respectively. Although hydroxyl radicals possess a slightly higher redox potential, sulfate radicals exhibit greater selectivity for electron-rich structures, such as aromatic rings and amine groups, making them particularly effective for degrading complex antibiotics and pharmaceutical pollutants. Persulfate is highly stable in the solid state and decomposes slowly in water, whereas PMS, due to its asymmetric structure, exhibits lower storage stability and decomposes more rapidly. The radical lifetimes also differ markedly: SO_4_˙^−^ persists for 30–40 µs, significantly longer than ˙OH (<1 µs), increasing the likelihood of productive pollutant oxidation rather than radical scavenging. Kinetically, PS requires a higher activation energy and is strongly dependent on external stimuli, such as heat, UV irradiation, or transition-metal catalysis, generating two sulfate radicals per molecule upon activation. PMS, in contrast, is more readily activated and produces both SO_4_˙^−^ and ˙OH almost instantaneously, enabling multiple oxidative pathways including hydrogen abstraction, electron transfer, and radical addition. Under neutral or alkaline conditions, SO_4_˙^−^ may further convert to ˙OH, expanding the oxidative versatility of both oxidants. The oxidizing power of radicals generated from persulfate (PS) and peroxymonosulfate (PMS) is a key factor governing their effectiveness in degrading persistent contaminants. The standard redox potential of the sulfate radical (SO_4_˙^−^) is typically reported in the range of 2.5–3.1 V, while the hydroxyl radical (˙OH) exhibits a redox potential of approximately 2.7–2.8 V in aqueous systems. In addition to their strong oxidizing ability, these radicals differ significantly in their lifetimes. The sulfate radical generally has a longer lifetime (30–40 µs) compared with the hydroxyl radical, whose lifetime is typically less than 1 µs, allowing sulfate radicals to participate more effectively in selective oxidation reactions with organic contaminants. These physicochemical properties play a crucial role in determining the oxidation pathways and degradation efficiencies observed in sulfate radical-based advanced oxidation processes.^30,31^ These distinctions, summarized in Table 1, comparative properties of persulfate (PS) and peroxymonosulfate (PMS), highlight that PDS is particularly suited for high-strength wastewater and for aromatic pollutants. In contrast, PMS offers broader applicability across a range of emerging contaminants, including pharmaceuticals.

**Table 1:** Comparative physicochemical properties of persulfate (PS) and monosulfate (PMS) relevant to radical generation in advanced oxidation processes

Properties	Persulfate PS	Peroxy monosulfate PMS	References
Main radicals	2SO_4_˙^−^	SO_4_˙^−^ + ˙OH	32–35
Activation energy	Higher	Lower	23 and 35–39
Stability	Very high	Moderate	40–42
Working pH range	2–11	3–10	43–46
Radical selectivity	Higher	Moderate (mixed radicals)	47–50
Suitability	High-strength wastewater, aromatic pollutants	Broad-range pollutants, including pharmaceuticals	51 and 52
Half-life	30–40 µs	<1 µs	50, 53 and 54
Redox potential *E*^0^	2.5–3.1 V	*E* ^0^ = 1.8 V	50

## 3. Activation techniques

**Fig. 2:**
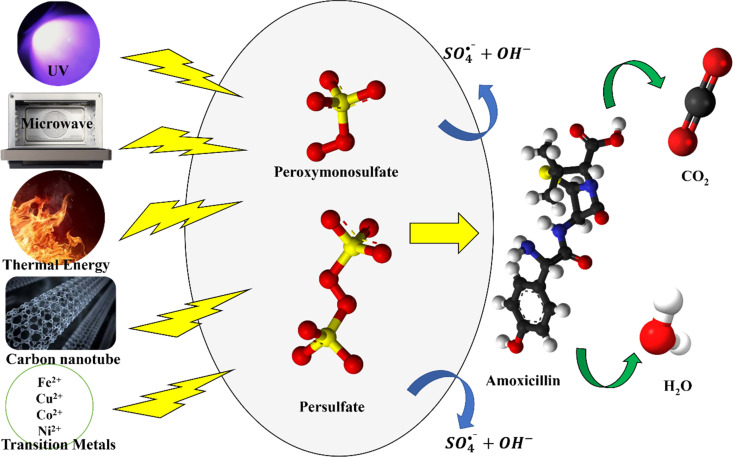
Overview of major activation strategies for persulfate (PS) and peroxymonosulfate (PMS) in sulfate radical-based advanced oxidation processes. The figure summarizes common activation pathways, including thermal activation, UV/visible-light irradiation, transition-metal catalysis, carbonaceous materials, and electrochemical/photoelectrocatalytic systems, highlighting their roles in generating sulfate radicals (SO_4_˙^−^) and hydroxyl radicals (˙OH) for the degradation of emerging contaminants.

Advanced oxidation processes (AOPs) based on hydroxyl radicals (˙OH) and sulfate radicals (SO_4_˙^−^) are currently considered effective methods for treating refractory antibiotics.^55^ Furthermore, SO_4_˙^−^ has shown excellent ability for the degradation of antibiotics since SO_4_˙^−^ has a higher oxidation potential (*E*^0^ = 2.5–3.1 V) and a longer lifetime (*t* = 30–40 µs) than that of ˙OH (*E*^0^ = 2.8 V, *t* = 0.02 µs).^56^ The activation of persulfate (PS, S_2_O_8_^2−^) and peroxymonosulfate (PMS, HSO_5_^−^) is essential for generating reactive sulfate (SO_4_˙^−^) and hydroxyl (˙OH) radicals that drive the degradation of pharmaceuticals such as amoxicillin. Various activation pathways have been developed to enhance radical yield, accelerate decomposition kinetics, and improve treatment efficiency under different environmental conditions. The oxidant persulfate, including peroxodisulfate (PS, S_2_O_8_^2−^) and peroxymonosulfate (PMS, HSO_5_^−^), can be employed to generate SO_4_˙^−^ in water under the presence of activators that may be ultraviolet (UV), ultrasound (US), heat, microwave (MW), transition metals (Fe^2+^, Cu^2+^, Co^2+^), and carbon materials (activated carbon, biochar).^23^ The corresponding activation mechanism is depicted in Fig. 2.

### 3.1 Thermal activation

Thermal activation is one of the simplest methods for initiating the decomposition of PS and PMS. During these activation processes, external energy exceeding the bond energy of peroxy bonds (140–213.3 kJ mol^−1^) is applied to break the peroxy bonds in PS or PMS, thereby generating sulfate or hydroxyl radicals.^23^ Typically, temperatures above 50–60 °C substantially increase decomposition rates, while higher temperatures (>80 °C) promote complete activation. Thermal activation is attractive as it does not introduce additional chemical reagents into the process, but it isenergy-intensive for large-scale wastewater treatment. It is most effective in high-strength industrial effluents where thermal energy is already available (*e.g.*, pharmaceutical manufacturing discharges), enhancing homolytic cleavage and generating reactive radicals^57^ according to:

3 S_2_O_8_^2−^ → 2SO_4_˙^−^

4 HSO_5_^−^ → SO_4_˙^−^ + OH^−^

Thermal methods are operationally straightforward but often limited by high energy consumption and reduced applicability in heat-sensitive wastewater matrices.^58,59^ Due to its higher activation energy, PS requires a higher temperature threshold compared to PMS.

### 3.2 UV and visible-light activation

UV activation of PS and PMS is highly efficient because both oxidants exhibit strong UV absorption in the 220–300 nm range.^60^ UV photons induce rapid cleavage of peroxo bonds, generating SO_4_˙^−^ and ˙OH radicals. In PMS, UV activation often yields mixed-radical systems with enhanced oxidation capacity. Visible-light activation is feasible when catalysts (*e.g.*, TiO_2_, BiVO_4_, g-C_3_N_4_) are introduced to harvest visible photons, thereby promoting electron transfer to PMS/PS. UV/PMS processes have demonstrated rapid degradation of β-lactam antibiotics, making them well-suited for treatment of pharmaceutical wastewater.^61^

### 3.3 Transition-metal activation

Transition metals significantly accelerate PS/PMS activation through redox cycling (M^*n*+^/M^*n*+1^), producing SO_4_˙^−^ radicals *via* electron transfer. Iron, cobalt, copper, manganese, and nickel are the most widely studied activators.^62^ Fe^2+^/Fe^3+^: economical and environmentally benign; Fe^2+^ reacts rapidly with PS/PMS to produce SO_4_˙^−.^^63,64^ Co^2+^: highly effective but limited by toxicity concerns. Cu^2+^: efficient for PMS activation *via* Cu^2+^/Cu^+^ cycling.^65–68^ Mn^2+^: often used in heterogeneous forms (MnO_2_, Mn_3_O_4_). Ni-based catalysts: provide robust activation but must be controlled due to metal leaching risks.^69,70^

Transition-metal activation exhibits high performance in degrading antibiotics such as amoxicillin, ciprofloxacin, and sulfamethoxazole, owing to strong electron-transfer interactions between metal ions and peroxo species. Transition-metal ions and metal oxides activate PS and PMS *via* one-electron transfer pathways, following:

5 Mn^+^ + S_2_O_8_^2−^ → M^*n*+1^ + SO_4_˙^−^ + SO_4_^2−^

Additionally, Fe^2+^, Co^2+^, and Cu^2+^ are the most widely studied for their strong catalytic activity and efficient redox cycling. Heterogeneous catalysts such as Fe_3_O_4_, Co_3_O_4_, MnO_2_, and NiFe layered double hydroxides offer advantages including stability, reusability, and reduced risk of metal leaching. Metal activation is particularly effective for antibiotics owing to strong electron-transfer interactions between the catalyst and the oxidant.

### 3.4 Carbonaceous material activation

**Fig. 3:**
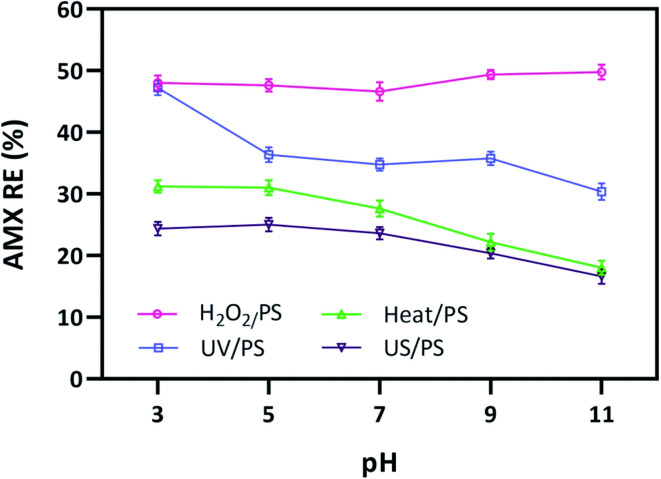
Effect of pH on PS activation in (a) H_2_O_2_/PS ([H_2_O_2_] = 1 mM, reaction time = 30 min), (b) UV/PS (reaction time = 15 min), (c) heat/PS (*T* = 60 °C, reaction time = 30 min), and (d) US/PS (US frequency = 30 kHz, reaction time = 30 min).^82^

Carbon-based materials, including biochar, activated carbon, graphene, reduced graphene oxide (rGO), and carbon nanotubes (CNTs), have emerged as sustainable, metal-free activators. Activation occurs through surface defects, carbon-bound functional groups, and persistent free radicals (PFRs), which enhance electron transfer to PS/PMS.^61,71^ Biochar and activated carbon demonstrate strong PMS activation capability, while graphene-derived materials exhibit enhanced conductivity, defect density, and catalytic activity for both PS and PMS.^71–73^ These materials are particularly attractive for antibiotic degradation because they offer low cost, tunable surface chemistry, and minimal secondary pollution. Carbon-based materials, including graphene derivatives, activated carbon, carbon nanotubes, and heteroatom-doped carbon frameworks, have emerged as efficient catalysts for the activation of persulfate and peroxymonosulfate due to their unique electronic structures and abundant surface defects. Unlike transition-metal-based catalysts that rely primarily on redox cycling, carbon materials typically activate PMS through electron transfer interactions between the delocalized π-electron network of the carbon surface and the oxidant molecule (Fig. 3).

The extended conjugated π-electron system in graphitic carbon facilitates charge redistribution, enabling PMS adsorption and subsequent activation by electron donation from the carbon lattice to the PMS peroxide bond (–O–O–). This interaction weakens the peroxide bond and promotes the generation of reactive oxygen species, such as sulfate radicals (SO_4_˙^−^), hydroxyl radicals (˙OH), and singlet oxygen (^1^O_2_). In addition, structural defects, edge sites, and heteroatom dopants (*e.g.*, N, S, or B) significantly modify the electronic density of carbon materials, thereby enhancing PMS activation efficiency.

Density Functional Theory (DFT) calculations have further confirmed that PMS molecules preferentially adsorb on defect-rich or heteroatom-doped carbon sites, where the adsorption energy is lower, and electron transfer is more favorable. These theoretical insights reveal that the activation mechanism often involves π-electron-mediated charge transfer, which can initiate both radical and non-radical oxidation pathways depending on the catalyst surface chemistry and reaction conditions. Consequently, tailoring the electronic structure of carbon materials through defect engineering or heteroatom doping has become an effective strategy for improving catalytic activity in sulfate radical-based advanced oxidation processes.

The activation of PS and PMS by carbon materials involves electron transfer. Both PS and PMS gain electrons to generate sulfate radicals:^74^

6 S_2_O_8_^2−^ + e^−^ → SO_4_˙^−^ + SO_4_^2−^

7 e^−^ + HSO_5_^−^ → SO_4_˙^−^ + OH^−^ or SO_4_˙^−^ + OH˙

### 3.5 Electrochemical and photoelectrocatalytic activation

Electrochemical activation relies on anodic oxidation or cathodic reduction to generate reactive species from PS and PMS. Electrochemical systems can continuously generate radicals in the absence of chemical additives, making them suitable for modern decentralized wastewater technologies. Anodic electrons reduce PMS or PS, forming SO_4_˙^−^, while the electrode simultaneously generates ˙OH through water oxidation.^75–77^

Photoelectrocatalytic activation further enhances radical production by combining UV/visible irradiation with anodic bias. This synergy enhances charge separation, increases the activation efficiency of PMS/PS, and significantly accelerates antibiotic degradation kinetics.

In addition to direct oxidative degradation, sulfate radical-based advanced oxidation processes may also promote radical coupling and polymerization pathways, particularly for phenolic or aromatic intermediates generated during oxidation reactions. Several recent studies have demonstrated that sulfate radicals (SO_4_˙^−^) can initiate radical–radical coupling reactions that lead to the formation of oligomeric or polymeric products rather than complete mineralization. These polymerized products generally exhibit lower solubility and higher molecular weight, which facilitates their partitioning onto catalyst surfaces or solid matrices. This phenomenon has been observed in persulfate-driven oxidation systems treating phenolic contaminants, where polymerization reactions contribute to pollutant removal by transforming dissolved compounds into recoverable solid-phase materials. Such processes may provide additional environmental benefits by reducing carbon mineralization to CO_2_ while enabling partial resource recovery through catalyst-associated polymer accumulation. Therefore, understanding the balance between oxidative degradation and polymerization pathways is important for optimizing sulfate radical-based treatment systems and for designing catalytic processes with improved sustainability and resource utilization.^78–81^

## 4. Degradation of amoxicillin

Amoxicillin (AMX) persists through conventional wastewater treatment and is increasingly treated *via* sulfate radical-based advanced oxidation processes (SR-AOPs) using persulfate (PS) and peroxymonosulfate (PMS). These processes generate reactive species, primarily sulfate radicals (SO_4_˙^−^) and hydroxyl radicals (˙OH), through thermal, photolytic, electrochemical, or catalytic activation, enabling transformation of AMX *via* β-lactam ring opening, aromatic hydroxylation, *N*-dealkylation, and further oxidation toward smaller acids and inorganic ions. Process performance depends on oxidant type/dose, pH, activation strategy, and matrix constituents that can quench or channel non-radical pathways.^50^

### 4.1 Process factors in SR-AOPs

Across sulfate radical-based AOPs, AMX typically exhibits apparent pseudo-first-order decay, with faster removal when oxidant and activation intensity are increased. For example, in PS systems activated by UV, AMX commonly reaches near-complete removal in tens of minutes with *k*_obs_ in the order of 10^−2^–10^−1^ min^−1^, reflecting efficient SO_4_˙^−^ generation under photon flux; comparable behavior is observed under thermal PS activation when the temperature is raised to accelerate radical formation and sustain *k*_obs_ in similar ranges. Ultrasound-activated PS often exhibits intermediate rates due to cavitation-assisted activation. In contrast, PS activation with co-feeding hydrogen peroxide can yield rapid initial AMX loss followed by slower tailing as radical demand from the matrix increases. PMS systems activated by transition-metal catalysts (*e.g.*, Co/Fe oxides or ferrites) frequently achieve faster apparent kinetics than non-activated PMS at the same dose due to concurrent SO_4_˙^−^ and ˙OH generation, and carbon-supported spinel ferrites (*e.g.*, CuNiFe_2_O_4_ on MWCNTs) have shown strong kinetic enhancement by promoting surface reactions and minimizing mass-transfer limitations.^82^

AMX degradation under SR-AOPs generally benefits from mildly acidic to neutral pH, where SO_4_˙^−^ is more stable and selective; at higher pH, sulfate radicals are converted to ˙OH, broadening reactivity but often raising scavenging losses (Fig. 1). In UV-PS systems, maintaining pH around 5–7 typically preserves SO_4_˙^−^ dominance and improves electron-transfer reactions with AMX's electron-rich sites; thermal PS activation similarly favors acidic-neutral conditions to curb radical hydrolysis and sustain effective *k*_obs_. Ultrasound-PS tends to show reduced sensitivity to pH swings but still performs best when bicarbonate/carbonate scavenging is limited, which is more readily achieved below neutral pH. PMS activated by Co/Fe catalysts often operates optimally near pH 6–7 to balance metal redox cycling with radical speciation; carbon–ferrite PMS catalysts (*e.g.*, MWCNTs–CuNiFe_2_O_4_) have demonstrated robust activity across neutral pH, leveraging mixed radical and surface-bound oxidants to maintain AMX removal in complex waters.^82^

Increasing oxidant concentration (PS/PMS) typically accelerates AMX decay until self-scavenging and background demand reduce marginal gains; strong activation strategies markedly lift *k*_obs_ by raising radical production rates. UV-PS shows a clear dose-response acceleration, with higher PS doses and irradiance increasing SO_4_˙^−^ flux; thermal PS provides similar improvements with temperature, and PS dosage is tuned accordingly. Ultrasound PS exhibits an activation-limited regime in which higher PS doses yield diminishing returns unless cavitation intensity is also increased. PMS with transition-metal catalysts (*e.g.*, Co/Fe oxides, spinel ferrites) responds strongly to oxidant dose up to a catalyst-specific optimum, after which radical recombination and scavenging dominate; PMS on conductive carbon supports (MWCNTs–CuNiFe_2_O_4_) sustains high activity by facilitating electron transfer and non-radical routes that help resist NOM and bicarbonate scavenging. In advanced PS photocatalytic systems (*e.g.*, layered double hydroxides or mixed-metal oxides), coupling light with tailored surfaces boosts activation efficiency. It broadens the usable oxidant window for rapid AMX removal.^82^

Catalyst selection shapes both the rate and the durability of AMX degradation in SR AOPs. Co-based catalysts efficiently activate PMS to produce mixed SO_4_˙^−^/˙OH, thereby driving rapid AMX decay under neutral pH. In contrast, Fe-based oxides offer lower-cost activation with careful control of leaching and redox cycling. Spinel ferrites (*e.g.*, CuNiFe_2_O_4_) supported on carbon nanotubes enhance PMS activation *via* conductive interfaces and abundant active sites, delivering high removal rates alongside improved resistance to matrix scavengers. For PS, UV and thermal activation reduce reliance on metals and simplify recovery, whereas photocatalytic PS activation with designed mixed-metal oxides (*e.g.*, Ba_2_CoMnO_5_) or LDH/CaCO_3_ architectures improves radical generation, supports non-radical pathways, and stabilizes performance in real waters. Doped carbons (N/S/B-doped) can activate both PS and PMS, enabling surface-bound oxidants and singlet oxygen routes that improve selectivity for AMX and mitigate radical quenching by halides and carbonate species.^82^

Under optimized SR-AOP conditions, activated PMS/PS, acidic-neutral pH, adequate oxidant dose, and robust catalysts, AMX is rapidly degraded with apparent pseudo-first-order behavior; achieving high rates requires balancing radical generation against matrix scavenging and selecting activation routes compatible with the water chemistry (*e.g.*, UV/heat for PS, Co/Fe/ferrite–carbon for PMS). In comparative studies, UV/heat/ultrasound activation of PS and PMS-catalyst systems consistently deliver near-complete AMX removal within minutes to tens of minutes, with faster kinetics observed when mixed radical and surface-bound oxidants are present and when pH and dose are tuned to limit scavengers (Table 2).^82^

**Table 2:** Summary of radical pathways and practical considerations in PS *vs.* PMS AOPs for amoxicillin

System	Dominant oxidant species	Typical pH window	Activation strategy	Kinetic range (*k*_obs_)	Practical notes
PS-UV	SO_4_˙^−^ (selective)	5–7	UV irradiation	10^−2^–10^−1^ min^−1^	Fast removal; metal-free; energy input required
PS-heat	SO_4_˙^−^ → ˙OH (with pH↑)	5–7	Thermal	10^−2^–10^−1^ min^−1^	Simple setup; watch self-scavenging at high PS
PS-ultrasound	SO_4_˙^−^ (cavitation-dependent)	∼6–7	Ultrasound	10^−3^–10^−2^ min^−1^	Cavitation intensity controls ates; matrix sensitive
PMS-Co/Fe	SO_4_˙^−^ + ˙OH (mixed)	6–7	Heterogeneous catalysis	10^−2^–10^−1^ min^−1^	High rates; manage leaching; robust at neutral pH
PMS-ferrite/MWCNT	Mixed + non-radical	6–7	Ferrite on carbon	10^−2^–10^−1^ min^−1^	Enhanced *via* conductive

### 4.2 Degradation rates and optimal conditions

**Fig. 4:**
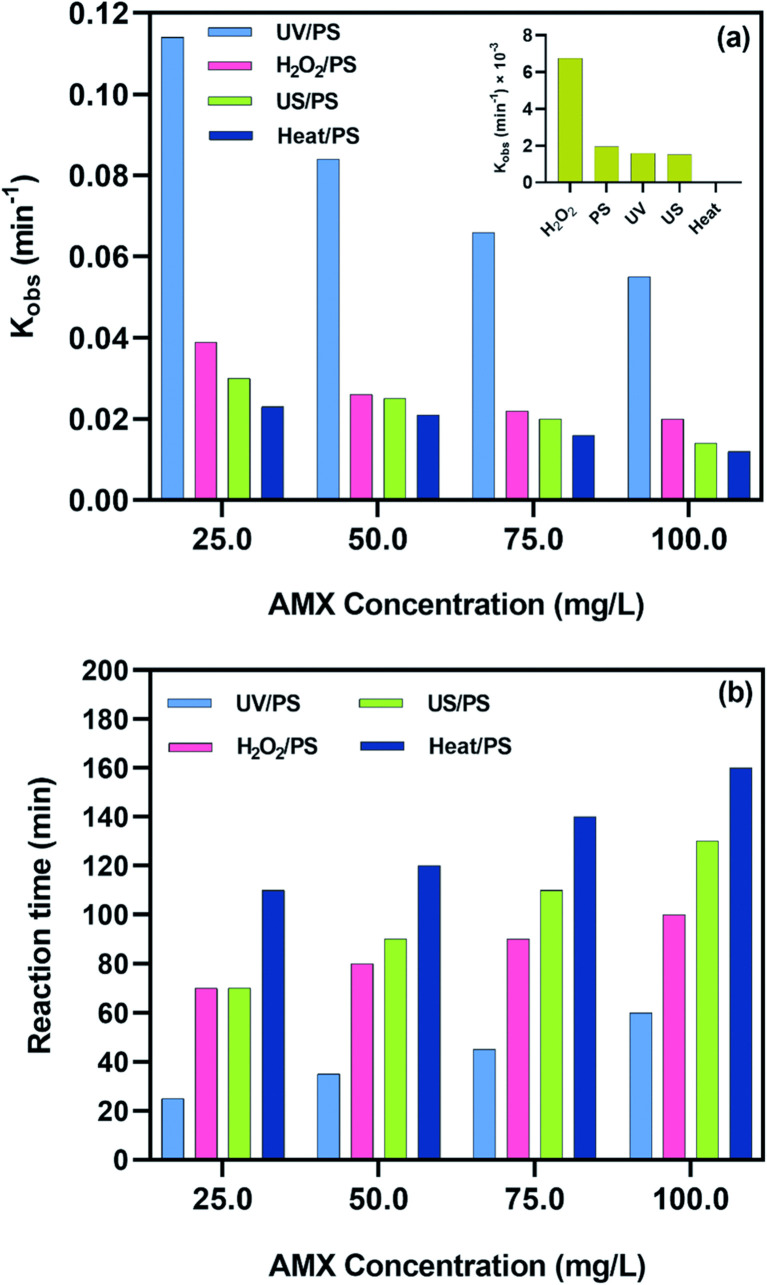
(a) Reaction rate constants obtained from the PFO kinetic model, and (b) time required for complete (100%) degradation of AMX using H_2_O_2_/PS, UV/PS, heat/PS, and US/PS processes.^82^

Representative operating windows and kinetic ranges (Fig. 4):

• PS-UV activation: rapid AMX removal with *k*_obs_ commonly in the 10^−2^–10^−1^ min^−1^ range under moderate PS doses and UV irradiance; optimal pH is mildly acidic to neutral to maintain SO_4_˙^−^ selectivity and minimize carbonate scavenging.^82^

• PS-heat activation: similar kinetic ranges achieved by increasing temperature (*e.g.*, moderate thermal activation) and PS dose, with optimal pH near 5–7 to preserve sulfate radicals and avoid rapid conversion to ˙OH unless broad reactivity is desired.^82^

• PS-ultrasound activation: intermediate *k*_obs_ due to cavitation-limited radical generation, performance improves with cavitation intensity, controlled pH, and PS dose up to the point of self-scavenging. Near-neutral pH reduces carbonate interference while retaining SO_4_˙^−^ stability.^82^

• PMS-metal catalysis (Co/Fe): high *k*_obs_*via* mixed radicals and surface reactions at neutral pH, with optimal PMS dose and catalyst loading tuned to limit recombination and leaching; carbon-supported ferrites further enhance rates by facilitating electron transfer and non-radical pathways.^83^

• Advanced PS photocatalysis (mixed-metal oxides/LDH): elevated rates through synergistic light activation and tailored catalytic surfaces; neutral pH and moderate PS doses are optimal for stability, with mechanistic evidence of multiple reactive oxygen species contributing to AMX degradation.^84^

Although numerous studies have demonstrated the high efficiency of persulfate (PS) and peroxymonosulfate (PMS) activation systems in degrading antibiotics and other emerging contaminants, most investigations are conducted in synthetic aqueous solutions under controlled laboratory conditions.^85–87^ In practical wastewater treatment, however, the presence of complex matrices can significantly influence the performance of sulfate radical-based advanced oxidation processes. Natural organic matter (NOM) and dissolved organic compounds can act as radical scavengers, competing with target pollutants and reducing degradation efficiency.^88–90^ Similarly, inorganic ions commonly present in wastewater, such as chloride (Cl^−^), bicarbonate (HCO_3_^−^), carbonate (CO_3_^2−^), and nitrate (NO_3_^−^), may either inhibit or modify radical reactions through scavenging or secondary radical formation. Variations in pH conditions may also affect the stability of PS and PMS and influence the interconversion between sulfate radicals (SO_4_˙^−^) and hydroxyl radicals (˙OH).^46,91,92^ Furthermore, catalyst fouling caused by the deposition of organic matter or inorganic precipitates can gradually decrease catalytic activity during long-term operation. Therefore, understanding these matrix effects is essential for selecting appropriate oxidant systems and designing efficient sulfate radical-based treatment technologies for real wastewater applications (Table 3).

**Table 3:** Influence of wastewater matrix components on the performance of persulfate (PS) and peroxymonosulfate (PMS) activation systems in advanced oxidation processes

Matrix factor	Effect on radical reactions	Impact on PS/PMS systems	Practical implication
Natural organic matter (NOM)	Radical scavenging	Reduced degradation efficiency	Higher oxidant dosage required
Chloride ions (Cl^−^)	Formation of reactive chlorine species	May enhance or inhibit oxidation	Depends on pollutant type
Bicarbonate/carbonate	Scavenging of SO_4_˙^−^ and ˙OH	Formation of less reactive radicals	Lower oxidation rates
pH variation	Influences radical transformation	Changes SO_4_˙^−^/˙OH ratio	Process optimization required
Catalyst fouling	Surface blockage	Reduced catalytic activity	Periodic regeneration needed

### 4.3 Pathways and toxicity of AMX in SR-AOPs

Amoxicillin degradation *via* PS and PMS-based AOPs proceeds through distinct oxidative pathways, generating key intermediates and requiring toxicity assessment to ensure environmental safety.

The degradation of AMX under SR-AOPs involves a series of oxidative transformations that dismantle its molecular structure. The initial and most critical step is the cleavage of the β-lactam ring, which is responsible for AMX's antimicrobial activity. SO_4_˙^−^ and ˙OH generated from activated PS or PMS attack the strained ring, leading to its opening and the formation of amoxicilloic acid, a less toxic, non-antibiotic intermediate.^83^

Following ring cleavage, aromatic hydroxylation occurs, particularly on the phenol moiety of AMX. Hydroxyl radicals are especially effective at adding hydroxyl groups to the aromatic ring, increasing the compound's polarity and facilitating further breakdown. For instance, PMS activated by CuNiFe_2_O_4_ nanoparticles supported on multi-walled carbon nanotubes has shown efficient hydroxylation, producing hydroxy-AMX derivatives detectable *via* LC-MS/MS.^83^ This step is crucial for destabilizing the molecule and preparing it for mineralization.

Another important pathway is *N*-dealkylation, where alkyl side chains attached to the amine group are removed. This reaction yields primary amines and aldehydes, which are further oxidized. PMS systems, especially those catalyzed by MnO_2_ or CoFe_2_O_4_, have demonstrated strong *N*-dealkylation activity under neutral pH conditions.^83^ These transformations are essential for reducing the molecular complexity of AMX and enhancing its biodegradability.

Thiazolidine ring oxidation is also observed, particularly in PS systems activated by heat or UV. The sulfur atom in the thiazolidine ring is oxidized to form sulfoxide and sulfone intermediates. These species are transient but reactive, contributing to the breakdown of AMX's core structure. Studies using UV-C-activated PS have confirmed the presence of these intermediates through FTIR and LC-MS analyses.^93^

The final stages of degradation involve side-chain cleavage and mineralization. Oxidative reactions break down the remaining molecular fragments into small organic acids such as oxalic and acetic acid, eventually leading to complete mineralization into CO_2_ and H_2_O. PMS systems with carbon-doped catalysts have achieved total organic carbon (TOC) removal rates exceeding 70% within 60 minutes, indicating effective mineralization.^83^

Toxicity assessment is a critical component of evaluating SR-AOPs. While AMX itself is toxic to aquatic organisms and contributes to antibiotic resistance, its degradation products must also be scrutinized. Bioassays using *Vibrio fischeri* luminescence inhibition and *Daphnia magna* immobilization have shown significant reductions in toxicity after treatment. For example, UV-C-activated PS reduced *Vibrio fischeri* inhibition from 80% to less than 10% post-treatment.^93^ Disk diffusion assays further confirmed the loss of antimicrobial activity following β-lactam ring cleavage.

However, some intermediates, such as aldehydes and sulfoxides, may exhibit transient toxicity. Therefore, comprehensive toxicity profiling using both chemical and biological methods is essential. PMS systems activated by MWCNTs–CuNiFe_2_O_4_ have demonstrated superior detoxification performance, with over 90% reduction in toxicity indicators and complete loss of antibiotic activity.^83^ In addition to experimental bioassays, computational toxicity prediction tools have become widely used for evaluating the potential environmental risks associated with transformation products generated during advanced oxidation processes. Quantitative structure-activity relationship (QSAR) models, such as Ecological Structure-Activity Relationships (ECOSAR) and the Toxicity Estimation Software Tool (T.E.S.T.), are frequently employed to estimate the acute and chronic toxicity of organic compounds toward aquatic organisms, including fish, daphnia, and algae. These predictive platforms use molecular descriptors and structural similarity algorithms to estimate toxicity endpoints, enabling rapid screening of degradation intermediates formed during persulfate or peroxymonosulfate oxidation.

Such computational approaches are particularly valuable when multiple intermediate products are generated, and experimental toxicity testing is impractical or time-consuming. By combining QSAR-based toxicity prediction with experimental bioassays, researchers can obtain a more comprehensive evaluation of the environmental safety of advanced oxidation processes. Consequently, integrating predictive modeling tools with laboratory toxicity assays is an effective strategy for assessing the ecological implications of antibiotic degradation and guiding the development of safer, more sustainable wastewater treatment technologies.

In summary, AMX degradation under PS- and PMS-based AOPs involves multiple oxidative pathways, each contributing to the molecule's breakdown and detoxification. The formation of specific intermediates and their subsequent transformation must be carefully monitored to ensure environmental safety and treatment efficacy.

## 5. Kinetic and mechanistic insights

The degradation of AMX in PS and PMS systems generally follows pseudo-first-order kinetics when the oxidant is present in excess. This kinetic model simplifies the reaction rate to depend only on AMX concentration, allowing direct comparison across activation methods (Table 2). For example, UV-activated PS systems have been reported *k*_obs_ between 0.05–0.12 min^−1^,^93^ while PMS activated by CoFe_2_O_4_ nanoparticles achieved higher values, up to 0.15 min^−1^ under neutral pH conditions.^83^ These findings highlight the importance of activation strategy in determining radical flux and effective reaction rates.

Mechanistically, AMX degradation is driven by highly reactive radicals, primarily SO_4_˙^−^ and ˙OH. Sulfate radicals are more selective and stable at acidic to neutral pH, favoring electron-rich sites such as aromatic rings and amino groups in AMX.^50^ In contrast, hydroxyl radicals exhibit broader reactivity, attacking a wide range of functional groups through hydrogen abstraction and hydroxylation. PMS-based systems often generate both radicals simultaneously, creating a mixed regime that accelerates degradation.^83^ PS systems activated thermally or photolytically, however, tend to favor sulfate radical pathways, especially under acidic to neutral conditions.

Experimental quenching studies provide strong evidence of radical selectivity. Methanol, which quenches both SO_4_˙^−^ and ˙OH, and *tert*-butanol, which selectively quenches ˙OH, are commonly used to differentiate pathways.^50^ Results consistently show that PMS-metal catalysis produces mixed radical activity, while UV-PS systems are dominated by sulfate radicals.^93^ This selectivity influences the formation of degradation intermediates: sulfate radicals promote β-lactam ring opening, while hydroxyl radicals enhance aromatic hydroxylation.

Different activation methods shape the mechanistic landscape of AMX degradation. Thermal PS activation generates sulfate radicals efficiently at elevated temperatures, with rate constants increasing exponentially with temperature.^50^ UV-PS activation produces radicals *via* photolysis of persulfate, offering rapid kinetics but requiring energy input. PMS-Co/Fe catalysis facilitates redox cycling of transition metals, yielding both sulfate and hydroxyl radicals under neutral pH. Carbon-supported ferrites, such as CuNiFe_2_O_4_/MWCNTs, enhance electron transfer and enable near-surface oxidation, improving resilience against radical scavengers in real wastewater.^83^

Matrix components such as natural organic matter (NOM), bicarbonate, and halides act as radical scavengers, reducing effective AMX degradation rates.^87^ To mitigate these effects, catalyst design has focused on enhancing surface-bound oxidation and promoting non-radical pathways. For example, PMS activated by nitrogen-doped carbon catalysts has demonstrated significant AMX removal even in NOM-rich matrices, suggesting that non-radical oxidation can complement radical pathways.^94^

Mechanistic confirmation in SR-AOPs typically involves a combination of quenching experiments, spectroscopic analysis, and identification of intermediates. Selective radical quenchers are used to distinguish between sulfate and hydroxyl radicals,^49^ while LC-MS/MS and FTIR provide insights into transformation products and changes in functional groups.^93^ Total organic carbon (TOC) measurements assess mineralization efficiency, and photoluminescence (PL) and electrochemical impedance spectroscopy (EIS) evaluate charge separation in photocatalytic systems.^95^ These diagnostic tools are essential for validating proposed mechanisms and optimizing treatment protocols. The kinetic and mechanistic characteristics of PS *vs.* PMS systems for AMX degradation are summarized in Table 4.

**Table 4:** Kinetic and mechanistic characteristics of PS *vs.* PMS systems in AMX degradation

Activation system	Dominant radical(s)	Typical *k*_obs_ (min^−1^)	Optimal pH	Mechanistic notes
UV-PS	SO_4_˙^−^	0.05–0.12	5–7	Selective ring opening; energy input required
Thermal-PS	SO_4_˙^−^ → ˙OH	0.04–0.10	5–7	Temperature-dependent radical yield
PMS-CoFe_2_O_4_	SO_4_˙^−^ + ˙OH	0.10–0.15	6–7	Mixed radical regime; high efficiency
PMS-MWCNTs/ferrite	SO_4_˙^−^ + ˙OH + non-radical	0.12–0.18	6–7	Enhanced electron transfer; scavenger-resistant
PMS-N-doped carbon	Non-radical + ˙OH	0.08–0.14	6–8	Effective in NOM-rich matrices

## 6. Comparison of PS *vs.* PMS systems

AOPs based on PS and PMS have emerged as powerful tools for degrading persistent pharmaceuticals like AMX. While both oxidants generate SO_4_˙^−^ and ˙OH under activation, their physicochemical properties, activation requirements, and environmental profiles differ significantly. A comparative evaluation of these systems is essential to guide their selection for specific treatment scenarios (Table 5).

**Table 5:** Comparative attributes of PS *vs.* PMS systems for AMX degradation

Attribute	Persulfate (PS)	Peroxymonosulfate (PMS)
Radical generation	Primarily SO_4_˙^−^*via* UV/thermal/electrochemical activation	Mixed SO_4_˙^−^ and ˙OH *via* catalytic activation
Degradation efficiency	High under-optimized activation; selective oxidation	Typically, faster due to the dual-radical mechanism
Activation energy	Higher; requires UV, heat, or electrochemical input	Lower; activated by metals, carbons, or light
Cost profile	Lower reagent cost; higher energy demand	Higher reagent cost; lower activation overhead
Environmental impact	Minimal secondary pollution in non-metal systems	Potential metal leaching requires recovery protocols
Catalyst options	UV, heat, LDH, mixed-metal oxides	Co/Fe oxides, spinel ferrites, doped carbons
pH sensitivity	Optimal in-neutral range	Effective near neutral; broader pH tolerance
Matrix resilience	Sensitive to scavengers; selective pathways	More robust in complex matrices; mixed mechanisms

In terms of degradation efficiency, PMS-based systems often outperform PS under similar conditions due to their ability to generate both SO_4_˙^−^ and ˙OH simultaneously.^87^ This dual-radical mechanism enhances the oxidation of AMX's diverse functional groups, including the β-lactam ring and aromatic moieties. PMS also exhibits faster activation kinetics, especially when catalyzed by transition metals like cobalt or iron. In contrast, PS requires more intensive activation, typically *via* UV light, heat, or electrochemical methods to achieve comparable radical yields.^50^ However, PS systems offer greater selectivity and stability under acidic to neutral pH, which can be advantageous in matrices with high scavenger content.^50^

Cost considerations also influence the choice between PS and PMS. PMS is generally more expensive per unit mass, but its higher reactivity and lower activation energy can reduce overall operational costs in some configurations.^96^ PS, while cheaper, may require additional energy input or catalyst loading to reach similar performance levels. Moreover, PMS systems activated by heterogeneous catalysts often face challenges related to metal leaching and catalyst recovery, thereby increasing long-term maintenance costs and environmental risks.

Activation energy is another critical factor.^96^ PMS has a lower activation threshold and can be efficiently triggered by a wide range of catalysts, including spinel ferrites and doped carbons. This flexibility allows PMS systems to operate effectively under ambient conditions. PS, on the other hand, demands higher energy input for activation, particularly in non-catalytic setups. However, recent advances in photocatalytic PS systems, such as those using layered double hydroxides or mixed-metal oxides, have improved activation efficiency and broadened the applicability of PS in low-energy contexts.

Environmental impact assessments reveal nuanced differences. PMS systems, especially those involving transition metals, may pose risks of secondary pollution due to metal leaching. Proper catalyst design and recovery protocols are essential to mitigate these effects.^87^ PS systems, particularly those activated by light or heat, avoid metal-related concerns but may generate secondary oxidants or byproducts that require monitoring.

Both PS and PMS systems exhibit strong capabilities for amoxicillin detoxification, yet their long-term viability hinges on factors such as water matrix complexity, catalyst durability, and downstream treatment needs. As summarized in Table 5, PMS-based approaches tend to deliver quicker and more versatile reactivity, whereas PS systems offer enhanced precision and stability, particularly under controlled activation conditions. Selecting the appropriate system should be based on site-specific considerations, including available energy sources, ease of catalyst recovery, and overall environmental compatibility.

## 7. Challenges and future perspectives

Despite the promising performance of PS and PMS-based AOPs for amoxicillin degradation, several practical barriers remain before these systems can be widely applied in real wastewater treatment. Key issues include catalyst durability and recovery, the complexity of scaling laboratory findings to full-scale operations, and the need for integration with complementary technologies to enhance efficiency and resilience. Addressing these challenges will be critical to ensuring that SR-AOPs evolve from experimental success to sustainable solutions in environmental practice.

### 7.1 Catalyst recovery and stability

One of the foremost challenges in SR-AOPs is the recovery and long-term stability of heterogeneous catalysts. Transition-metal-based catalysts, such as CoFe_2_O_4_, MnO_2_, and CuNiFe_2_O_4_, are widely used to activate PMS and PS due to their high redox activity and ability to generate reactive radicals. However, repeated use often leads to metal leaching, structural degradation, and loss of catalytic activity. This not only reduces process efficiency but also raises environmental concerns due to secondary pollution. Strategies such as immobilizing catalysts on conductive supports (*e.g.*, multi-walled carbon nanotubes, graphene oxide) or embedding them in porous matrices (*e.g.*, zeolites, biochar) have shown promise in enhancing catalyst durability and facilitating recovery.^83,94^ Future research should focus on designing recyclable, low-leaching catalysts with high surface area and stable redox cycling to ensure sustainable operation in real wastewater conditions.

In addition to conventional catalyst supports, several advanced immobilization strategies have recently emerged to mitigate metal leaching and improve catalyst recyclability. Magnetic catalysts based on Fe_3_O_4_ or spinel ferrites enable rapid separation using external magnetic fields, significantly reducing catalyst loss during operation. Encapsulation of active metals within carbon frameworks or metal-organic frameworks (MOFs) has also shown promising results by stabilizing active sites and preventing metal dissolution. Furthermore, the development of single-atom catalysts and defect-engineered carbon materials offers a promising pathway for maximizing catalytic activity while minimizing metal loading and environmental risks. Future studies should focus on designing robust catalyst architectures that maintain high catalytic activity while ensuring long-term stability and minimal metal leaching in complex wastewater environments.

### 7.2 Scaling up SR-AOPs for real wastewater treatment

Translating laboratory-scale SR-AOPs to full-scale wastewater treatment remains a significant hurdle. Most studies on AMX degradation are conducted in controlled environments using synthetic solutions, which do not accurately reflect the complexity of real wastewater matrices. Real effluents contain a diverse mix of organic matter, inorganic ions, and competing contaminants that can quench reactive radicals or interfere with catalyst performance. Additionally, operational parameters such as flow rate, residence time, and reactor design must be optimized for continuous treatment. Pilot-scale demonstrations are needed to evaluate the feasibility of SR-AOPs under dynamic conditions. Integration with existing treatment infrastructure, such as coupling with biological processes or membrane systems, may offer a pathway to scalable implementation.^50,87^ Addressing these challenges will require interdisciplinary collaboration between chemists, engineers, and environmental scientists.

In addition to pilot-scale studies, future research should focus on developing continuous-flow reactor configurations, such as fluidized-bed reactors, catalytic membrane reactors, and packed-bed systems, which offer improved mass transfer and enhanced catalyst utilization. Process modeling and techno-economic analysis will also be essential for evaluating the feasibility of large-scale SR-AOP implementation. Optimizing operational parameters such as oxidant dosage, hydraulic retention time, and catalyst loading will help minimize operational costs and energy consumption while maintaining high pollutant removal efficiency.

### 7.3 Integration with adsorption or membrane filtration

To enhance the overall efficiency and selectivity of AMX removal, SR-AOPs can be integrated with adsorption or membrane filtration technologies. Adsorption using activated carbon, biochar, or metal-organic frameworks (MOFs) can pre-concentrate AMX and reduce the load on oxidation systems. This hybrid approach also helps mitigate radical scavenging by natural organic matter. Similarly, membrane filtration, such as ultrafiltration or nanofiltration, can retain AMX and its degradation intermediates, allowing for targeted oxidation in a confined volume. However, membrane fouling and permeability loss remain technical challenges, especially when dealing with complex wastewater. Future research should explore synergistic designs, such as catalytic membranes or adsorbent-oxidant composites, that combine physical separation with chemical degradation.^95,96^ These integrated systems could offer higher removal rates, lower energy consumption, and improved resilience against matrix variability.

Recently, catalytic membrane systems have attracted increasing attention as an efficient platform for integrating separation and oxidation processes. In these systems, catalytic materials are immobilized directly onto membrane surfaces, allowing simultaneous pollutant filtration and PMS/PS activation. This configuration not only enhances degradation efficiency but also reduces membrane fouling by degrading organic contaminants at the membrane interface. Additionally, coupling SR-AOPs with energy-efficient processes such as electrochemical activation powered by renewable energy sources may further improve the sustainability and scalability of these systems. Future research should therefore focus on optimizing hybrid treatment configurations that combine catalytic oxidation, membrane separation, and adsorption to achieve high treatment efficiency with reduced operational costs.

## 8. Conclusion

Sulfate radical-based advanced oxidation processes (SR-AOPs) have emerged as highly effective and versatile technologies for degrading amoxicillin and other persistent antibiotics in aquatic environments. Compared with conventional hydroxyl radical-based oxidation systems, persulfate (PS) and peroxymonosulfate (PMS) activation produce sulfate radicals with longer lifetimes, greater selectivity toward electron-rich organic compounds, and broader operational pH ranges. These characteristics make sulfate radical systems particularly suitable for degrading complex pharmaceutical pollutants that are resistant to conventional treatment methods.

This review provides a comprehensive and systematic comparison of PS- and PMS-based activation strategies, including thermal, photochemical, transition-metal, carbonaceous material, electrochemical, and photoelectrocatalytic activation routes. By integrating mechanistic insights with process performance data, this work highlights the differences in radical generation pathways, reaction kinetics, and operational conditions that govern degradation efficiency. In particular, persulfate systems demonstrate strong potential for treating high-strength and aromatic-rich wastewaters due to their high stability and ability to generate multiple sulfate radicals, whereas PMS systems offer rapid activation and versatile oxidation pathways under relatively mild conditions.

Beyond summarizing existing research, this review advances the current understanding of SR-AOPs by providing a critical comparison of activation technologies in terms of energy efficiency, catalytic performance, and environmental sustainability. Special attention is given to the relevance of energy consumption metrics such as electrical energy per order (EE/O) as indicators for evaluating the practical feasibility of different activation systems. In addition, the review highlights the importance of understanding the relationship between operational parameters, degradation pathways, and the potential toxicity of intermediate products, which remains a crucial factor in assessing the environmental safety of oxidation processes.

Despite the significant progress achieved in PS/PMS-based oxidation systems, several challenges remain before these technologies can be widely implemented at an industrial scale. Catalyst deactivation, metal ion leaching, energy demand, and the incomplete mineralization of transformation products continue to limit long-term operational stability. Future research should therefore focus on the development of stable heterogeneous catalysts with minimal metal leaching, energy-efficient reactor configurations, and integrated hybrid treatment systems such as adsorption–oxidation or catalytic membrane processes. Furthermore, systematic evaluation of transformation product toxicity and pilot-scale studies under realistic wastewater conditions will be essential to ensure the safe and sustainable deployment of SR-AOP technologies.

Overall, the insights provided in this review help bridge the gap between fundamental catalytic mechanisms and practical environmental applications, offering guidance for the rational design of next-generation sulfate radical-based oxidation systems to effectively control antibiotic pollution.

## Conflicts of interest

The author declares that there are no conflicts of interest regarding the publication of this paper. We wish to confirm that there are no known conflicts of interest associated with this publication and that no significant financial support has been received for this work that could have influenced its outcome.

## Data Availability

No primary research results, software or code have been included and no new data were generated or analysed as part of this review.
